# MT1-MMP Cooperates with TGF-β Receptor-Mediated Signaling to Trigger SNAIL and Induce Epithelial-to-Mesenchymal-like Transition in U87 Glioblastoma Cells

**DOI:** 10.3390/ijms222313006

**Published:** 2021-11-30

**Authors:** Souad Djediai, Narjara Gonzalez Suarez, Layal El Cheikh-Hussein, Sahily Rodriguez Torres, Loraine Gresseau, Sheraz Dhayne, Zoé Joly-Lopez, Borhane Annabi

**Affiliations:** 1 Laboratoire d’Oncologie Moléculaire, Université du Québec à Montréal, C.P. 8888, Succ. Centre-ville, Montreal, QC H3C 3P8, Canada; djediai.souad@courrier.uqam.ca (S.D.); gonzalez_suarez.narjara@courrier.uqam.ca (N.G.S.); el_cheikh-hussein.layal@courrier.uqam.ca (L.E.C.-H.); rodriguez_torres.sahily@courrier.uqam.ca (S.R.T.); gresseau.loraine@courrier.uqam.ca (L.G.); 2 Département de Chimie, and CERMO-FC, Université du Québec à Montréal, Montreal, QC H3C 3P8, Canada; dhayne.sheraz@courrier.uqam.ca (S.D.); joly-lopez.zoe@uqam.ca (Z.J.-L.)

**Keywords:** glioblastoma, MT1-MMP, EGCG, EMT, concanavalin A, SNAIL, STAT3

## Abstract

Epithelial-to-mesenchymal transition (EMT) recapitulates metastasis and can be induced in vitro through transforming growth factor (TGF)-β signaling. A role for MMP activity in glioblastoma multiforme has been ascribed to EMT, but the molecular crosstalk between TGF-β signaling and membrane type 1 MMP (MT1-MMP) remains poorly understood. Here, the expression of common EMT biomarkers, induced through TGF-β and the MT1-MMP inducer concanavalin A (ConA), was explored using RNA-seq analysis and differential gene arrays in human U87 glioblastoma cells. TGF-β triggered SNAIL and fibronectin expressions in 2D-adherent and 3D-spheroid U87 glioblastoma cell models. Those inductions were antagonized by the TGF-β receptor kinase inhibitor galunisertib, the JAK/STAT inhibitors AG490 and tofacitinib, and by the diet-derived epigallocatechin gallate (EGCG). Transient gene silencing of MT1-MMP prevented the induction of SNAIL by ConA and abrogated TGF-β-induced cell chemotaxis. Moreover, ConA induced STAT3 and Src phosphorylation, suggesting these pathways to be involved in the MT1-MMP-mediated signaling axis that led to SNAIL induction. Our findings highlight a new signaling axis linking MT1-MMP to TGF-β-mediated EMT-like induction in glioblastoma cells, the process of which can be prevented by the diet-derived EGCG.

## 1. Introduction

One of the compelling reasons that makes it difficult to foresee brain cancer therapy response relates to the adaptive metabolic mechanisms that regulate their chemoresistance phenotype [1,2]. Among the therapy resistance mechanisms that regulate cancer cell death/survival balance, the fundamental importance of epithelial-to-mesenchymal transition (EMT) in metastasis and progression of cancer has been recognized [3]. Interestingly, transforming growth factor (TGF)-β is a well-known contributor to EMT, and TGF-β downstream signaling was found to be highly active in high-grade glioblastomas, the most fatal tumor of the central nervous system, where elevated TGF-β activity was associated with poor clinical outcome [4]. The tumor-promoting function of TGF-β is therefore a promising potential therapeutic target in high-grade gliomas, including glioblastoma multiforme (GBM) [5].

Recent analysis of transcriptomic datasets about mesenchymal shift in GBM show that, in terms of epithelial and mesenchymal phenotype, the majority of GBM appear to have a transcriptomic profile that is more mesenchymal than epithelial [6]. If induced, this phenotype can be shifted toward an even more mesenchymal phenotype in an EMT-like process in glioma cells [7]. A better understanding of the molecular regulation of the EMT during tumor spreading will therefore help design better therapeutics to target this program when treating GBM. Among the various signaling pathways associated with glioma malignancy, TGF-β signaling is hypothesized to be directly involved in such molecular mechanisms [8,9]. A comparative proteome mapping of the U87 human glioblastoma cell line, with and without TGF-β treatment, identified numerous proteins involved in the molecular mechanisms of GBM oncogenesis and TGF-β signaling [10]—among which, increases in 512 proteins upon TGF-β treatment were associated with survival, proliferation, cell migration, and DNA repair. Moreover, studies have reported that TGF-β is able to induce metastatic processes and tumor progression via autocrine mechanisms [11,12].

TGF-β is a multifunctional cytokine that acts as a downstream signaling molecule both in the early stages of tumorigenesis as a potential tumor suppressor [13] and then as a tumor-promoting factor promoting EMT and tumor metastasis through Smad and Smad-independent signaling pathways [14]. The activation of the classical Smad signaling pathway occurs when TGF-β first binds to the extracellular segment of TGF receptor-type II that leads to the phosphorylation of TGF receptor-type I. This then phosphorylates and activates the downstream Smads for intracellular signaling [15]. Recently, dietary-derived anthocyanidins have been shown to inhibit EMT through a TGFβ/Smad2 signaling pathway in glioblastoma cells [16]. In addition, the suppressing effects of green tea extract and epigallocatechin-3-gallate (EGCG) on TGF-β-induced EMT were reported in human cervical cancer cells [17]. Whether the pleiotropic actions of dietary polyphenols also target other EMT-mediated cues or downstream signaling pathways such as signal transducer and activator of transcription 3 (STAT3) in EMT [18] remains unknown.

Among specific brain cancer biomarkers promoting invasion and metastasis and characterized by both matrix metalloproteinase (MMP) catalytic functions and intracellular signaling properties, membrane type-1 matrix metalloproteinase (MT1-MMP), a key membrane-bound MMP, is involved in extracellular matrix (ECM) degradation [19,20,21] and, more recently, signal transducing functions leading to angiogenesis [22], autophagy [23,24], inflammation [25,26], immune response [27], and cell death processes [28,29]. Interestingly, type I collagen, a major MT1-MMP substrate in the ECM, is a powerful inducer of cell-surface MT1-MMP expression through TGF-β–Smad signaling [26,30,31]. Moreover, EGCG was found to inhibit MT1-MMP-mediated downstream signaling involving STAT3 [32,33].

In the current study, we investigated the possible crosstalk between TGF-β signaling and MT1-MMP in the setting of EMT-like processes in an established U87 grade IV human glioblastoma cell model. Our findings help better characterize the pleiotropic actions of EGCG on processes regulating the invasive phenotype of brain cancer cells and on the novel crosstalk between MT1-MMP and TGF-receptor-mediated signaling in EMT.

## 2. Results

### 2.1. SNAIL among the Common EMT Biomarkers Induced by Concanavalin A and TGF-β in U87 Glioblastoma Cells

**Figure 1:**
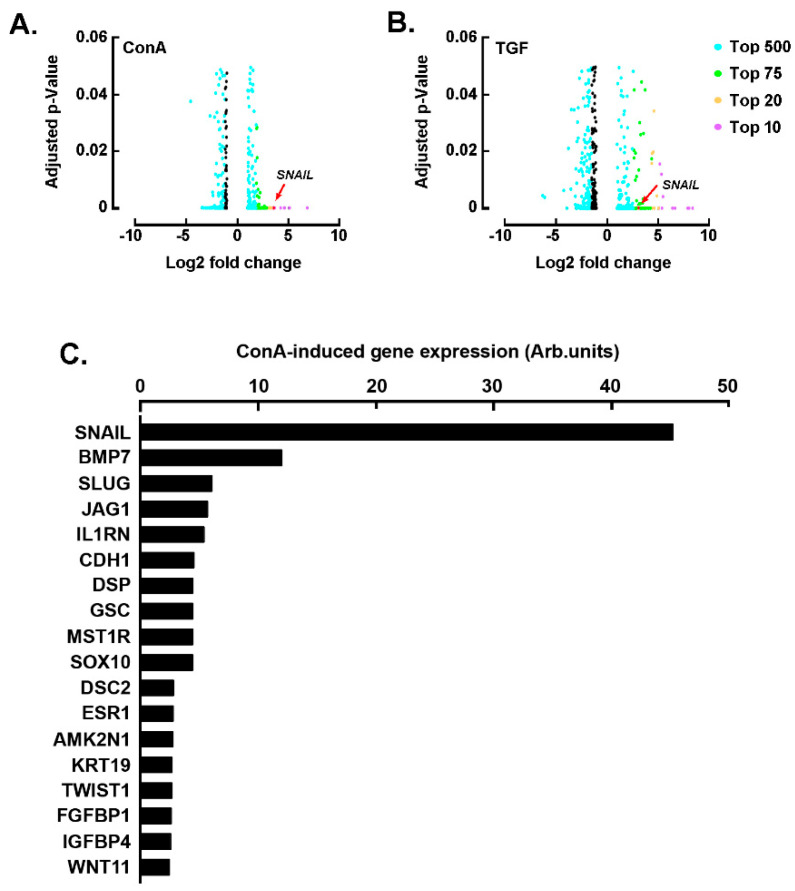
Concanavalin A- and TGF-β-mediated transcriptional control of SNAIL. Volcano plots of differentially expressed genes (DEGs) between U87 glioblastoma cells treated or not with (**A**) 30 μg/mL of concanavalin A (ConA) for 24 h and (**B**) U87 glioblastoma cells treated or not with 10 nM TGF-β. The adjusted *p*-value is shown on the y-axis and log2 fold change is plotted in the x-axis. Colored points indicate genes called as DEGs at adjusted *p*-value ≤ 0.05, and the different colors indicate the top upregulated or downregulated genes, according to the legend. Three replicates for untreated cells, two replicates for ConA-treated cells and two replicates for TGF-β-treated cells. (**C**) Total RNA was extracted from U87 glioblastoma cells treated or not with ConA, and RT-qPCR was performed using a RT2-Profiler gene array to assess EMT gene expression levels. Ratios of ConA-induced gene expression on vehicle-treated cells gene expression are expressed from one representative experiment out of two.

Concanavalin A (ConA) has been demonstrated to trigger numerous signaling pathways in glioblastoma cells, which among other cellular processes, lead to pro-angiogenic and pro-inflammatory events [34]. ConA has also been shown to require MT1-MMP-mediated signaling to trigger U87 glioblastoma cell invasive phenotype [35]. Here, we wanted first to assess whether ConA could also trigger epithelial-to-mesenchymal (EMT) biomarkers expression and second to compare ConA to the classical EMT inducer TGF-β. We therefore isolated total RNA from U87 glioblastoma cells treated with either vehicle, 10 nM TGF-β, or 30 μg/mL ConA, and performed RNA sequencing (RNA-seq) analysis to identify genes potentially involved in ConA- and TGF-mediated response. A gene expression PCA plot mapping the distances between samples revealed major differences for two replicates (ConA3 and TGF4), which were then discarded from future analysis (Supplemental Figure S1). Differentially expressed genes (DEGs) analysis between ConA-treated cells and untreated cells and between TGF-treated cells and untreated cells was performed. Among the DEGs identified in ConA-treated cells, SNAIL (ENSG00000124216) was found among the top 10 most upregulated genes, with a ~10.5-fold change (*p*-value 8.03 × 10^−13^) (Figure 1A, Supplemental Table S1). SNAIL was also upregulated in cells treated with TGF-β, with a fold change of ~9.9 (*p*-value 4.45 × 10^−10^) (Figure 1B, Supplemental Table S2). Whereas TGF-mediated transcriptional regulation of SNAIL in EMT is well recognized [36], the mechanisms linking SNAIL regulation and ConA induction of EMT are less understood and were further confirmed using a differential gene array approach. In agreement with the RNA-seq analysis, SNAIL was also found among the most significantly upregulated genes (Figure 1C).

### 2.2. U87-Derived Neurospheres Response to TGF-β Can Be Inhibited by EGCG

**Figure 2:**
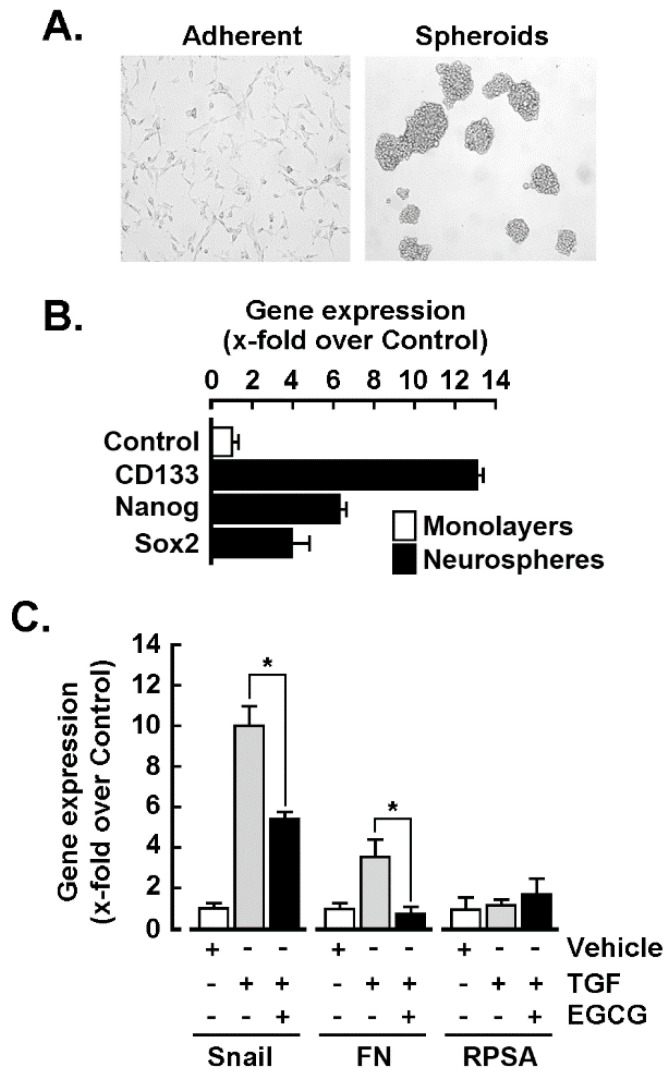
U87-derived neurospheres response to TGF-β can be inhibited by EGCG. (**A**) U87 glioblastoma neurospheres were cultured as described in the Materials and Methods section, and representative phase contrast pictures were taken. Spheroids were treated or not with 10 nM TGF-β or in combination with 30 μM EGCG for 18 h; total RNA extracted and RT-qPCR analyses were performed to assess (**B**) cancer stemness and (**C**) EMT gene expression levels. Probability values of less than 0.05 were considered significant and an asterisk identifies such significance.

2D monolayer U87 glioblastoma cells were cultured into 3D spheroid-forming conditions (neurospheres, Figure 2A), as these are known to be much closer to the tumor than adherent cells and to recapitulate some cancer stem cell phenotype as well as including increased expression of CD133, Nanog, and Sox2 (Figure 2B). Response to TGF-β was next assessed similarly as in the monolayers condition, and EMT markers SNAIL and fibronectin were found to be significantly increased, whereas the ribosomal protein SA (RPSA) expression remained unaltered (Figure 2C, grey bars). Interestingly, TGF-β-mediated inductions of SNAIL and fibronectin were reduced by 30 μM EGCG (Figure 2C, black bars).

### 2.3. Galunisertib and EGCG Inhibit TGF-β- and Concanavalin A-Mediated SNAIL Induction

**Figure 3:**
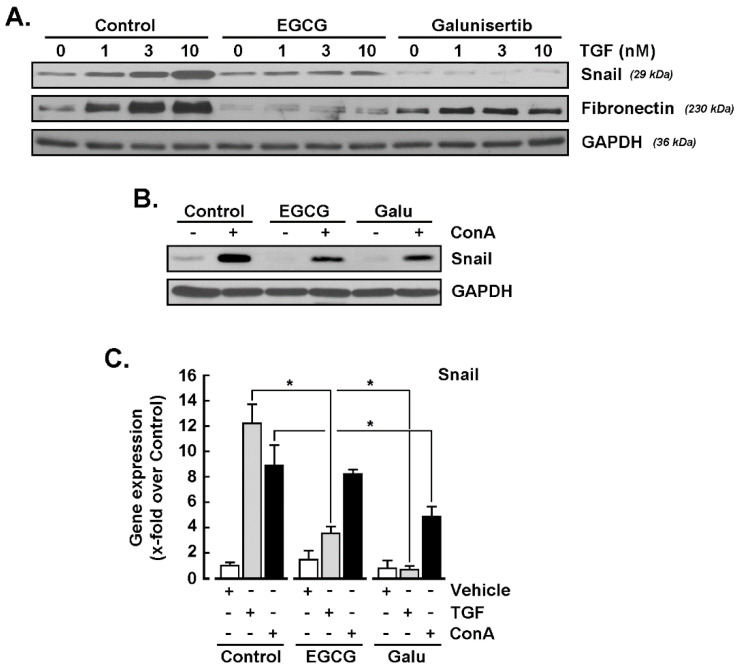
SNAIL induction by concanavalin A involves a TGF-β receptor-mediated signaling axis component. (**A**) Serum starved U87 glioblastoma cells were treated with increasing concentrations of TGF-β in the presence or not of 30 μM EGCG, or 10 μM galunisertib for 24 h. SNAIL, fibronectin and GAPDH protein expression were then assessed by immunoblotting using the respective cell lysates. (**B**) Serum-starved U87 glioblastoma cells were treated with 30 μg/mL of concanavalin A (ConA) for 24 h in the presence or not of 30 μM EGCG or 10 μM galunisertib (Galu) for 24 h. SNAIL and GAPDH protein expression were then assessed by immunoblotting using the respective cell lysates. (**C**) Total RNA was extracted from treated cells, and RT-qPCR was performed to assess SNAIL gene expression as described in the Materials and Methods section. Data are means ± SD from three independent experiments. Probability values of less than 0.05 were considered significant and an asterisk identifies such significance.

Given the ConA-mediated induction of SNAIL, and that SNAIL is a strong downstream biomarker also induced upon TGF-β treatment, we next explored whether any common signaling crosstalk was involved between TGF-β-mediated signaling and the effects of ConA. Serum-starved U87 glioblastoma cells were treated with increasing concentrations of TGF-β in the presence or absence of either 30 μM EGCG or 10 μM galunisertib, a selective TGF-β receptor type I (TGF-βRI) kinase inhibitor [37]. The protein expression levels of SNAIL, fibronectin, and GAPDH were then assessed by immunoblotting using cell lysates. SNAIL was effectively induced upon TGF-β treatment and found to be inhibited by EGCG and by galunisertib (Figure 3A, upper panel). While the expression of GAPDH remained unaltered (Figure 3A, lower panel), that of fibronectin was also repressed by both agents, suggesting the involvement of common signaling intermediates in the induction of EMT biomarkers (Figure 3A, middle panel). In agreement with its increased transcript levels (Figure 1), SNAIL was also found induced by ConA, but it was inhibited in the presence of either EGCG or galunisertib (Figure 3B). Finally, total RNA was extracted from treated cells, and RT-qPCR was performed as described in the Materials and Methods section. SNAIL induction was again confirmed in TGF-β- and ConA-treated cells at the transcript level, whereas it was inhibited in both cases by galunisertib (Figure 3C). On the other hand, EGCG was unable to inhibit ConA-induced SNAIL gene expression, whereas it inhibited SNAIL induction in TGF-β-treated cells (Figure 3C).

### 2.4. MT1-MMP Silencing Represses TGF-β- and Concanavalin A-Mediated Induction of SNAIL

**Figure 4:**
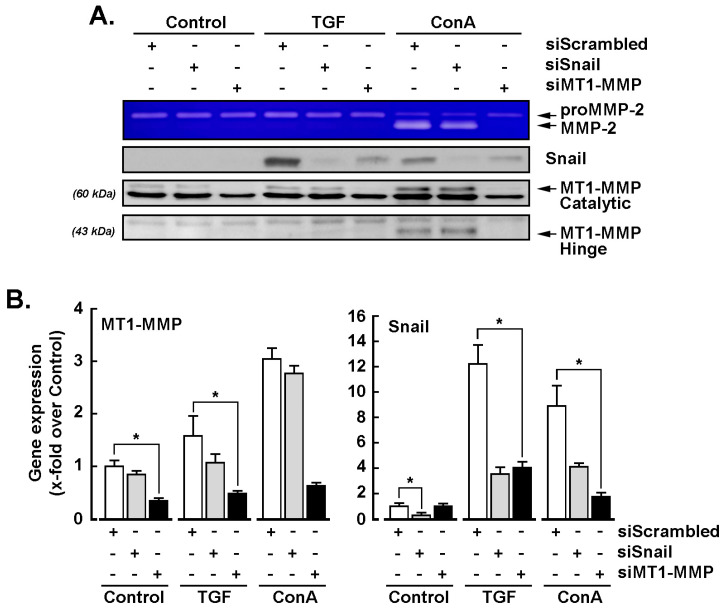
MT1-MMP silencing represses TGF-β- and concanavalin A-mediated induction of SNAIL. Transient siRNA-mediated gene silencing was performed in U87 glioblastoma cells transfected with siScrambled, siSNAIL, and siMT1-MMP. Serum-starved cells were next treated with 10 nM TGF-β or 30 μM ConA for 24 h. (**A**) The conditioned media were harvested and subjected to gelatin zymography to assess proMMP-2 activation into MMP-2, whereas cell lysates were subjected to immunoblotting of SNAIL and MT1-MMP catalytic and hinge forms for protein expression. (**B**) Total RNA was also extracted from the respective above conditions, and RT-qPCR was performed to monitor MT1-MMP and SNAIL gene expression. Data are means ± SD from three independent experiments. Probability values of less than 0.05 were considered significant and an asterisk identifies such significance.

SiRNA-mediated gene silencing was performed in U87 glioblastoma cells transiently transfected with siScrambled, siSNAIL, or siMT1-MMP, as described in the Materials and Methods section. Serum-starved cells were next treated with 10 nM TGF-β or 30 μM ConA for 24 h and conditioned media harvested to assess proMMP-2 activation status by gelatin zymography. MT1-MMP silencing was confirmed, as both ConA-mediated proMMP-2 activation and MT1-MMP proteolytic hinge domain formation were abrogated (Figure 4A). SNAIL silencing efficacy was also confirmed, as neither TGF-β nor ConA were able to upregulate its expression (Figure 4A). Total RNA was extracted from the respective conditions and RT-qPCR was performed to monitor MT1-MMP and SNAIL gene expression levels. We observed that, in cells where MT1-MMP was silenced, neither TGF-β nor ConA were able to induce SNAIL (Figure 4B, right panel), whereas in cells that were silenced for SNAIL, MT1-MMP transcript levels remained unaltered upon TGF-β or ConA treatments (Figure 4B, left panel).

### 2.5. Concanavalin A and TGF-β Trigger Common Signaling Pathways

**Figure 5:**
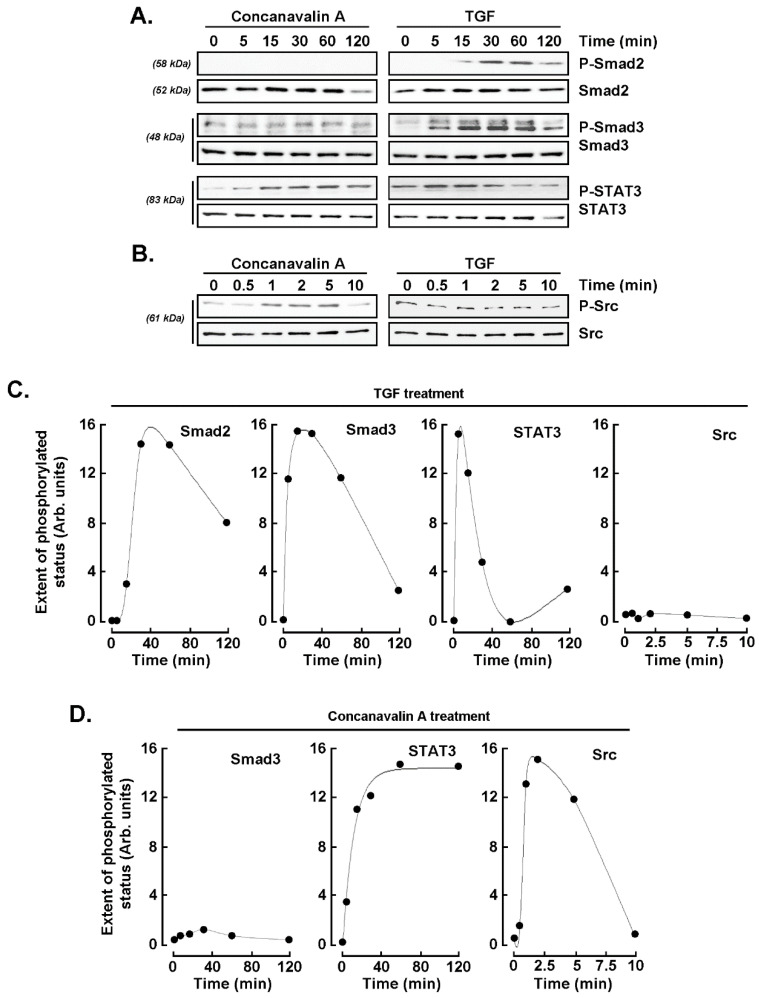
Concanavalin A and TGF-β share common signaling axis. U87 glioblastoma cells were treated with 10 nM TGF-β or 30 μM ConA for the indicated times, and cell protein lysates were harvested. Long time-course was performed from 0 to 120 min in (**A**) to monitor Smad2, Smad3, and STAT3 protein status, whereas a short time-course was performed from 0 to 10 min in (**B**) to monitor Src phosphorylation status. Scanning densitometric analysis was performed of a representative experiment for (**C**) TGF-β treatment and (**D**) concanavalin A treatment.

As both TGF-β and ConA appear to upregulate SNAIL expression, we next investigated whether TGF-β-mediated signaling pathways were also involved in ConA induction of SNAIL. U87 glioblastoma cells were therefore treated with TGF-β or ConA for various time-courses, and cell protein lysates were harvested. Long time-course (0–120 min) was performed to monitor Smad2, Smad3, and STAT3 protein phosphorylation status (Figure 5A), whereas a shorter time-course (0–10 min) was performed in order to monitor Src phosphorylation status (Figure 5B). Scanning densitometric analysis of representative Western blots revealed transient increases in Smad2/3 and STAT3 phosphorylation upon TGF-β treatment peaking between 15 and 30 min, whereas no increases in Src phosphorylation were found (Figure 5C). On the other hand, in ConA-treated cells, no evidence of Smad2 phosphorylation was found, and only slight increases in Smad3 phosphorylation were observed, whereas significant sustained phosphorylation of STAT3, as well as transient Src phosphorylation, was found (Figure 5D).

### 2.6. Evidence for MT1-MMP and SNAIL Involvement in the Chemotactic Response to TGF-β in U87 Glioblastoma Cells

**Figure 6:**
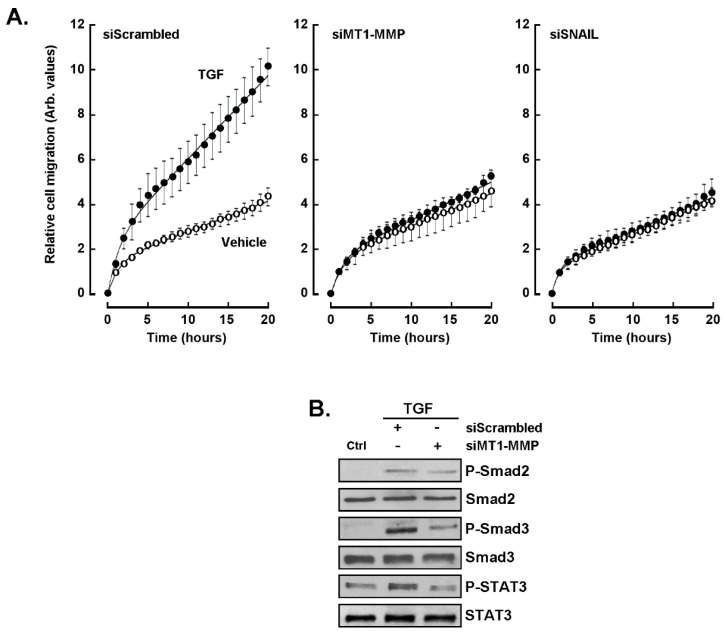
Evidence for MT1-MMP and SNAIL involvement in the chemotactic response of U87 glioblastoma cells to TGF-β. siRNA-mediated gene silencing was performed in U87 glioblastoma cells transiently transfected with siScrambled, siSNAIL, and siMT1-MMP. (**A**) Cell chemotaxis was next assessed in unstimulated (vehicle, open circles) or in response to TGF-β (closed circles) as described in the Materials and Methods section. (**B**) Cells where MT1-MMP was silenced (siMT1-MMP) were treated with 10 nM TGF-β for 30 min, and cell protein lysates were harvested to monitor Smad2, Smad3, and STAT3 phosphorylation status.

We next wished to elucidate the potential crosstalk between TGF-β receptor-mediated signaling and that of ConA-induced MT1-MMP. Gene silencing was thus performed in U87 glioblastoma cells transiently transfected with siScrambled, siSNAIL, and siMT1-MMP siRNAs, and cell chemotaxis was assessed in unstimulated (vehicle) or in response to TGF-β as described in the Materials and Methods section. Chemotaxis was significantly induced in response to TGF-β in siScrambled-transfected cells (Figure 6A, left panel). In contrast, silencing of either MT1-MMP or SNAIL abrogated the chemotactic response to TGF-β (Figure 6A, middle and right panels). Whether MT1-MMP silencing abrogated the phosphorylation status of those TGF-β-signaling intermediates examined above was next explored. The global phosphorylation status of Smad2/3 and of STAT3 were significantly reduced in cells where MT1-MMP was silenced (Figure 6B). This suggests that an MT1-MMP-mediated signaling crosstalk exists with TGF-β-receptor-mediated signaling.

### 2.7. Pharmacological Inhibition of the STAT3 Signaling Pathway Abrogates the Chemotactic Response to TGF-β

**Figure 7:**
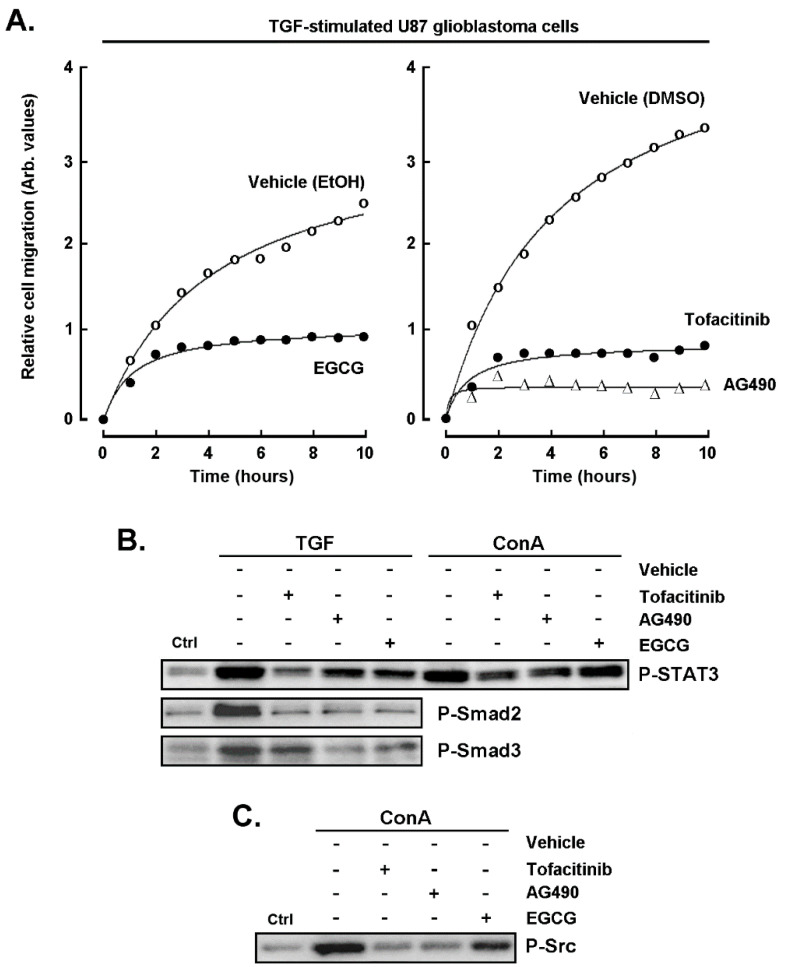
Pharmacological inhibition of the STAT3 signaling pathway abrogates the chemotactic response to TGF-β. (**A**) U87 glioblastoma cell chemotaxis was assessed in the presence of 10 nM TGF-β, in the presence or not of 30 μM EGCG (left panel, vehicle ethanol), or tofacitinib, AG490 (right panel, vehicle DMSO). (**B**) Cells were treated for 30 min with 10 nM TGF-β or 30 μM ConA, and cell protein lysates were harvested to monitor Smad2, Smad3, and STAT3 phosphorylated status. (**C**) Cells were treated for 5 min with 30 μM ConA, and cell protein lysates were harvested to monitor Src phosphorylated status.

Given the above evidence that STAT3 may link MT1-MMP signaling to that of TGF-β, and that SNAIL appeared to be a common intermediate in ConA and TGF-β responses, we next assessed the impact of AG490 and of tofacitinib, two JAK/STAT3 inhibitors [38,39], as well as of EGCG [32,40] on the TGF-β-induced chemotactic response. U87 glioblastoma cells migration was found induced with TGF-β (Figure 7A; vehicle condition), whereas pharmacological inhibitors of STAT3, including AG490, tofacitinib, and EGCG, collectively prevented that increase. Such reduced chemotactic response is in part explained through the reduced phosphorylation status of STAT3 in both TGF-β- and ConA-stimulated cells (Figure 7C), as well as of Src in ConA-stimulated cells (Figure 7C). Furthermore, alternative cell migration (wound healing) assay was performed and confirmed that both galunisertib and EGCG abrogated TGF-β-mediated healing (data not shown).

## 3. Discussion

Epithelial-to-mesenchymal transition (EMT) is a mechanism associated with tumor progression, invasion, and metastasis, in which polar epithelial cells eventually transfer to mesenchymal phenotype cells [41]. Accordingly, sustained elevation of SNAIL was found to promote glial–mesenchymal transition after irradiation in malignant glioma [42]. However, although EMT may be a common pattern in glioma progression, the therapeutic interventions affecting the occurrence and magnitude of EMT during the clinical course of GBM still remains a constant challenge [43,44], as the microenvironment that induces EMT in gliomas is characterized by hypoxia and the enrichment of myeloid cells following stimulation by TGF-β [45]. Meanwhile, there is a transformation process similar to EMT during the progress of GBM, which is called EMT-like process [6,7]. EMT-like process mainly represents the decrease in epithelial markers such as E-cadherin and the increase in interstitial markers such as N-cadherin and vimentin. Studies thus far demonstrate that the overexpression of a “mesenchymal” gene expression signature is related to the poor prognosis of glioma patients, indicating that the EMT-like process is closely related to the invasive phenotype of GBM [6].

It has been previously reported that TGF-β levels are high in the blood serum and tumor tissue of patients with malignant glioma and that this level was correlated with the type of malignancy, the tumor developmental stage, and the patient prognosis [10]. Here, we show that a crosstalk between MT1-MMP and TGF-β-receptor signaling regulates TGF-β-mediated EMT-like induction of SNAIL expression and chemotactic response in a model of GBM through, in part, STAT3. Moreover, the oncogenic contribution of MT1-MMP in GBM invasion is not only strictly controlled through the extent of its expression levels but also controlled through its signal transducing capacity [46,47,48]. In addition to initiating carcinoma cell invasion, TGF-β-induced EMT-like process can also guide cancer cells to then de-differentiate and gain cancer stem cell-like properties. EMT also allows the generation of stromal cells that support and instruct cancer progression. As such, it is inferred that EMT, cancer stem cells (CSCs), and drug resistance form the lethal “three combinations” and become the main barrier for glioma to be cured. Here, we show that 3D neurospheres exhibited the CSC phenotype and were responsive to TGF-β-induced EMT signaling, and that this was inhibited by EGCG (Figure 2). The inhibition of EMT may thus prevent invasion and metastasis of tumor cells, reduce CSCs, and overcome drug resistance. Accordingly, acquisition of EMT has been documented in CSC, and phytochemicals; in particular curcumin, EGCG, sulforaphane, resveratrol, and genistein have been shown to interfere with intrinsic CSC pathways in vitro and in human xenograft mice, leading to elimination of CSC [49].

During the EMT process, malignant cells start to intravasate into the surrounding blood vessels in order to migrate to other parts of the body. To accomplish this process, the ECM and basement membrane of blood vessels have to be degraded by MMPs [50]. Whereas the most relevant MMPs in this invasive process are MMP-2 and MMP-9 [51], SNAIL was found to induce MMP-9 expression, and EMT was found to be necessary for intravasation of lymph vessels in GBM and other cancers [52]. EMT has been shown to cooperate with MMP activity in GBM, allowing cells to gain access to lymph vessels. Preliminary data suggest this new EMT-associated drug target, in combination with stereotactic radiosurgery, may provide a potential rationale for future treatments [53]. Moreover, recent evidence supports such crosstalk, as TGF-β facilitated MT1-MMP-mediated proMMP-9 activation and invasion in an oral squamous cell carcinoma cell model [54]. Moreover, MT1-MMP-mediated proprotein maturation of TGF-β1, accelerating the release of free TGF-β1 in type II airway epithelial cells A549, and was found to induce EMT [55].

TGF-β activates the JAK/STAT pathway via the induction of leukemia inhibitory factor (LIF) secretion acting through an autocrine/paracrine loop [56]. After binding of LIF to its cell surface receptor LIFR, heterodimerization with another transmembrane protein, glycoprotein-130 (gp130), occurs, followed by recruitment of JAK and STAT3 via Src-homology-2 (SH2) domains in the LIFR-gp130 heterodimer. STAT3 can induce the expression of Sox2 stimulating self-renewal capacity and stemness in glioma-derived CSCs [4]. JAK/STAT3 signaling was also found to be required for TGF-β-induced EMT in lung cancer cells [57]. Intriguingly, and although TGF-β-mediated signaling was found active in our U87 glioblastoma cell model, no significant phosphorylation of Src was observed in our hands, which contrasts with other reports and suggests cell type-specific signaling [58].

## 4. Materials and Methods

### 4.1. Materials

Sodium dodecylsulfate (SDS), epigallocatechin-3-gallate (EGCG), tofacitinib, AG490, and bovine serum albumin (BSA) were purchased from Sigma (Oakville, ON, Canada). Electrophoresis reagents were from Bio-Rad (Mississauga, ON, Canada). The enhanced chemiluminescence (ECL) reagents were from Amersham Pharmacia Biotech (Baie d’Urfé, QC, Canada). Micro bicinchoninic acid protein assay reagents were purchased from Pierce (Rockford, IL, USA). Galunisertib (LY2157299) was from MedChemExpress (Monmouth Junction, NJ, USA). The polyclonal antibody against the MT1-MMP hinge domain was from Chemicon (Temecula, CA, USA). The polyclonal antibodies against SNAIL, fibronectin, Smad2/3, phosphorylated Smad2/3, Src, phosphorylated Src, STAT3, and phosphorylated STAT3 were obtained from Cell Signaling Technology Inc. (Danvers, MA, USA). Horseradish peroxidase-conjugated donkey anti-rabbit and anti-mouse IgG secondary antibodies were obtained from Jackson ImmunoResearch Laboratories (West Grove, PA, USA). The monoclonal antibody against glyceraldehyde 3-phosphate dehydrogenase (GAPDH) was from Advanced Immunochemical Inc. (Long Beach, CA, USA). Horseradish peroxidase-conjugated donkey anti-rabbit and anti-mouse IgG secondary antibodies were from Jackson ImmunoResearch Laboratories (West Grove, PA, USA).

### 4.2. Cell Culture

Human U87 glioblastoma cells were purchased from American Type Culture Collection (ATCC; Manassas, VA, USA). Serum starvation was performed by culturing the cells in Eagle’s minimal essential medium (EMEM; Gibco BRL, Grand Island, NY, USA) and 100 units/mL penicillin/streptomycin, and from which the 10% inactivated fetal bovine serum (Hyclone Laboratories, Logan, UT, USA) was removed. Spheroids formation was carried out in low adhesion 24-well plates (Corning Costar, Corning, NY, USA) and incubated for 3 days before quantification. Spheroids were defined as rounded aggregates of cells with a smooth surface and poor cell-to-cell definition.

### 4.3. Immunoblotting Procedures

Human U87 glioblastoma cells were lysed, and proteins were separated by SDS–polyacrylamide gel electrophoresis (PAGE). In order to detect MT1-MMP proteolytic processing, samples were subjected to SDS-PAGE gels under reducing conditions. After electrophoresis, proteins (30 μg) were electrotransferred to polyvinylidene difluoride membranes, which were then blocked for one hour at room temperature with 5% non-fat dry milk in Tris-buffered saline (150 mM NaCl, 20 mM Tris-HCl, pH 7.5) containing 0.3% Tween-20 (TBST; Bioshop, TWN510-500, Burlington, ON, Canada). Membranes were further washed in TBST and incubated with the primary antibodies (1/1000 dilution) in TBST containing 3% BSA and 0.1% sodium azide (Sigma-Aldrich, Oakville, ON, Canada, S2002), followed by a one-hour incubation with horseradish peroxidase-conjugated donkey anti-rabbit IgG at 1/2500 dilutions in TBST containing 5% nonfat dry milk. Immunoreactive material was visualized by enhanced chemiluminescence (Amersham Pharmacia Biotech, RPN3004).

### 4.4. Gelatin Zymography

Gelatin zymography was used to assess the extracellular levels and activation states of secreted proMMP-2 and MMP-2 activities. Briefly, an aliquot (20 μL) of the culture medium was subjected to SDS-PAGE in a gel containing 0.1 mg/mL gelatin (Sigma-Aldrich, Oakville, ON, Canada, G2625). The gels were then incubated in 2.5% Triton X-100 (Bioshop, TRX506.500) and rinsed in nanopure distilled water. Gels were further incubated at 37 °C for 20 h in 20 mM NaCl, 5 mM CaCl_2_, 0.02% Brij-35, and 50 mM Tris–HCl buffer, pH 7.6, and then stained with 0.1% Coomassie Brilliant blue R-250 (Bioshop, CBB250) and destained in 10% acetic acid, 30% methanol in water. Gelatinolytic activity was detected as unstained bands on a blue background.

### 4.5. Total RNA Isolation, cDNA Synthesis, and Real-Time Quantitative PCR

Total RNA was extracted from 10^7^ human U87 glioblastoma cell monolayers in 1 mL TRIzol™ as recommended by the manufacturer (Life Technologies, Gaithersburg, MD, USA). For cDNA synthesis, one μg of total RNA was reverse-transcribed into cDNA using a high-capacity cDNA reverse transcription kit (Applied Biosystems, Foster City, CA, USA). Gene expression was quantified by real-time quantitative PCR using iQ SYBR Green Supermix (BIO-RAD, Hercules, CA, USA). DNA amplification was carried out using an Icycler iQ5 (BIO-RAD, Hercules, CA, USA), and product detection was performed by measuring binding of the fluorescent dye SYBR Green I to double-stranded DNA. The following primer sets were provided by QIAGEN (Valencia, CA, USA): MT1-MMP (HS_MMP14_1_SG, QT00001533), SNAIL (Hs_SNAI1_1_SG, QT00010010), RPSA (Hs_RPSA_1_SG, QT00044310), CD133 (Hs_PROM1_1_SG, QT00075586), NANOG (Hs_NANOG_2_SG, QT01844808), SOX2 (Hs_SOX2_1_SG, QT00237601), PPIA (Hs_PPIA_4_SG, QT01866137), GAPDH (Hs_GAPDH_1_SG, QT00079247), and β-Actin (Hs_Actb_2_SG, QT01680476). The relative quantities of target gene mRNA were normalized against internal PPIA, GAPDH, and β-actin RNA and were measured by following a ΔCT method employing an amplification plot (fluorescence signal vs. cycle number). The difference (ΔC_T_) between the mean values in the triplicate samples of target gene and those of β-actin RNA was calculated by CFX manager Software version 2.1 (Bio-Rad) and the relative quantified value (RQV) was expressed as 2^−ΔC_T_^.

### 4.6. Total RNA Library Preparation and Sequencing

Total RNA (500 ng) was used for library preparation. RNA quality control was assessed with the Bioanalyzer RNA 6000 Nano assay on the 2100 Bioanalyzer system (Agilent technologies), and all samples had a RIN above 8. Library preparation was carried out with the KAPA mRNAseq Hyperprep kit (KAPA, Cat no. KK8581). Ligation was made with Illumina dual-index UMI (IDT), and 10 PCR cycles were required to amplify cDNA libraries. Libraries were quantified by QuBit and BioAnalyzer DNA1000. All libraries were diluted to 10 nM and normalized by qPCR using the KAPA library quantification kit (KAPA; Cat no. KK4973). Libraries were pooled to equimolar concentrations. Three biological replicates were generated. Sequencing was performed with the Illumina Nextseq500 using the Nextseq High Output 75 (1 × 75 bp) cycles kit. Around 15–20 M single-end PF reads were generated per sample. Library preparation and sequencing was performed at the Genomic Platform of the Institute for Research in Immunology and Cancer (IRIC) (Montreal, QC, Canada).

### 4.7. Reads Alignment and Differential Expression Analysis

Reads were 3′ trimmed for quality and adapter sequences using Trimmomatic version 0.35, and only reads with at least 50 bp in length were kept for further analyses. Trimmed reads were aligned to the reference human genome version GRCh38 (gene annotation from Gencode version 37, based on Ensembl 103) using STAR version 2.7.1a [59]. Gene expressions were obtained both as read count directly from STAR as well as computed using RNA-Seq by Expectation Maximization (RSEM) [60] in order to obtain normalized gene and transcript-level expression, in TPM values, for these stranded RNA libraries. Differential expression analysis was performed using DESeq2 version 1.22.2 [61]. The Package limma [62] was used to normalize expression data, and read counts data were analyzed using DESeq2. Principal component analysis (PCA) for the first two most significant components was conducted with R packages [63] found in iDEP (integrated Differential Expression and Pathway) analysis [64]. iDEP was also used to determine significant differentially expressed genes (DEGs) with DESeq2 with FDR adjusted p-value of 0.05 and fold-change with a cutoff of two.

### 4.8. Human EMT PCR Array

The RT^2^ Profiler^TM^ PCR Array for human epithelial-to-mesenchymal transition (EMT) (PAHS-090Z) was used according to the manufacturer’s protocol (QIAGEN). The detailed list of the key genes assessed can be found on the manufacturer’s website (https://geneglobe.qiagen.com/us/product-groups/rt2-profiler-pcr-arrays (accessed on 14 October 2021)). Using real-time quantitative PCR, we reliably analyzed expression of a focused panel of genes related to EMT biomarkers. Relative gene expressions were calculated using the 2^−ΔΔC_T_^ method, in which C_T_ indicates the fractional cycle number where the fluorescent signal reaches detection threshold. The “delta–delta” method uses the normalized ΔC_T_ value of each sample, calculated using five endogenous control genes (*B2M*, *HPRT1*, *RPL13A*, *GAPDH*, and *ACTB*). Fold change values are then presented as average fold change = 2(average ^ΔΔC_T_^) for genes in differentiated adipocytes relative to pre-adipocytes. Detectable PCR products were obtained and defined as requiring <35 cycles. The resulting raw data were then analyzed using the PCR Array Data Analysis Template (http://www.sabiosciences.com/pcrarraydataanalysis.php (accessed on 14 October 2021)). This integrated web-based software package automatically performs all ΔΔC_T_ based fold-change calculations from the uploaded raw threshold cycle data.

### 4.9. RNA Interference

U87 glioblastoma cells were transiently transfected with siRNA sequences using Lipofectamine-2000 transfection reagent (Thermo Fisher Scientific, Waltham, MA, USA). Gene silencing was performed using 20 nM siRNA against MT1-MMP (HS_Mmp14_6 HP siRNA, S103648841), SNAIL (Hs_SNAI1_5 HP siRNA, SI02636424), or scrambled sequences (AllStar Negative Control siRNA, 1027281). The above small interfering RNA and mismatch siRNA were all synthesized by QIAGEN and annealed to form duplexes.

### 4.10. Statistical Data Analysis

Data are representative of three or more independent experiments. Statistical significance was assessed using Student’s unpaired t-test. Probability values of less than 0.05 were considered significant and an asterisk identifies such significance in the figures.

## 5. Conclusions

**Figure 8:**
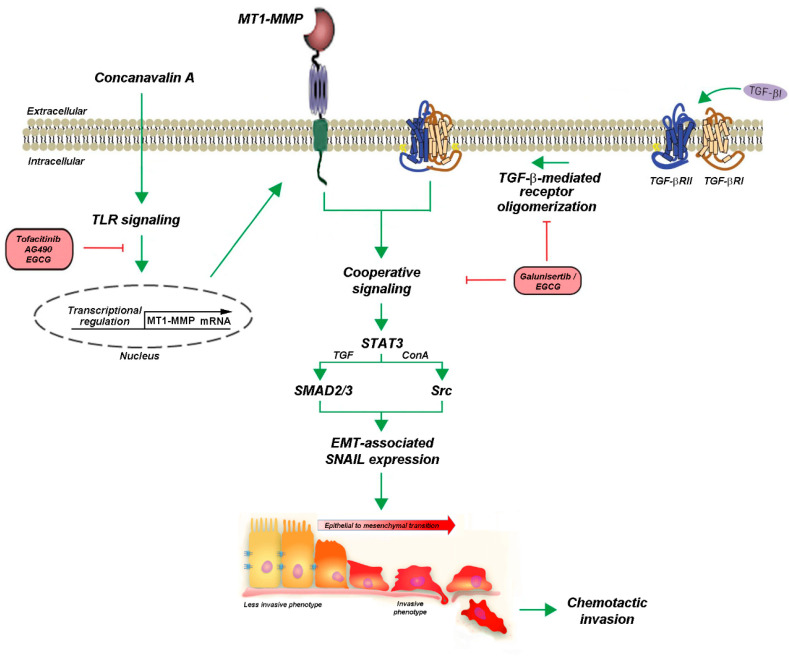
Scheme summarizing the cooperative signaling and pharmacological targeting between MT1-MMP and TGF-β receptor in U87 glioblastoma cells. We postulate that the MT1-MMP signal transducing activity cooperates with that of the TGF-β receptor in U87 glioblastoma cells. Concanavalin-A triggers MT1-MMP transcription and protein expression, possibly involving Toll-like receptors (TLR) signaling [32,46]. EGCG efficiently inhibits TGF-β-mediated EMT (SNAIL expression) by either competing with the TGF-β receptor binding site or by inhibiting TGF-β receptor oligomerization. Evidence for the common involvement of JAK/STAT signaling is provided through the pharmacological actions of tofacitinib and AG490. The acquisition of a less invasive to a more invasive phenotype leads to increased cell chemotactic invasive phenotype. These EMT-like events reflect the metastatic and chemoresistant phenotype that can ultimately be prevented by the diet-derived EGCG.

In summary, a cooperative signal transducing role for MT1-MMP was previously documented to take part in the transcriptional control of inflammasome-related genes [25], autophagy [24], and COX-2 induction [35]. Here, we now provide the first evidence for a cooperative crosstalk linking TGF-β receptor-mediated signaling to that of MT1-MMP, both activities of which have also been occurring in CSC [65,66,67]. It becomes tempting to suggest that TGF-β signaling may connect with some CSC phenotype in GBM, as CSCs represent a subset of GBM cells thought to be responsible for tumor initiation, progression, and relapse of disease [68]. Following the current crosstalk of TGF-β signaling and the underlying mechanisms identified here linking MT1-MMP, the promise of TGF-β targeted therapy or chemopreventive approaches, such as through diet-derived EGCG in malignant gliomas, is appealing (Figure 8). Several drugs targeting TGF-β signaling have been developed that have shown potent antitumor activity in preclinical models. A number of agents are currently being evaluated in early clinical studies in glioma patients, with the promise of TGF-β-targeted therapy. Here, we further provide evidence for such a chemopreventive diet-derived intervention that could be achieved through EGCG.

## Data Availability

All data generated or analyzed during this study are included in this published article.

## Supplementary Materials

The following are available online at https://www.mdpi.com/article/10.3390/ijms222313006/s1.

## Abbreviations

ConA	Concanavalin A
CSC	Cancer stem cells
DEG	Differentially expressed genes
EMT	Epithelial-to-mesenchymal transition
ECM	Extracellular matrix
EGCG	Epigallocatechin gallate
GBM	Glioblastoma multiforme
LIF	Leukemia inhibitory factor
MMP	Matrix metalloproteinase
MT1-MMP	Membrane type-1 matrix metalloproteinase
STAT3	Signal transducer and activator of transcription 3
TGF	Transforming growth factor
