# Supplementary material for: MT1-MMP Cooperates with TGF-β Receptor-Mediated Signaling to Trigger SNAIL and Induce Epithelial-to-Mesenchymal-like Transition in U87 Glioblastoma Cells
Source: Int J Mol Sci. 2021 Nov 30;22(23):13006. doi: 10.3390/ijms222313006 (PMC8657819; doi:10.3390/ijms222313006)
Supplement: Supplementary file 1 [file ijms-22-13006-s001.zip › ijms-1449984-supplementary/ijms-1449984-supplementary.pdf]

Supplementary

Fig.S1

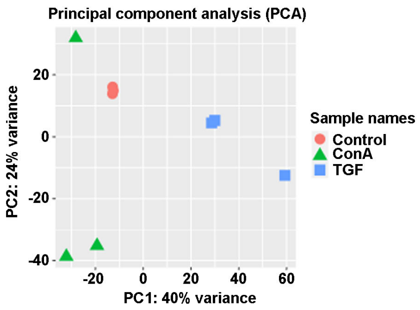

Figure S1. Principal component analysis (PCA) showing the variance for the two fist principal components for three replicates for TGF-treated cells (TGF), ConA-treated cells (ConA) and untreated cells (Control).
